# Herba Cistanches: Anti-aging

**DOI:** 10.14336/AD.2017.0720

**Published:** 2017-12-01

**Authors:** Ningqun Wang, Shaozhen Ji, Hao Zhang, Shanshan Mei, Lumin Qiao, Xianglan Jin

**Affiliations:** ^1^Department of Traditional Chinese Medicine, Xuanwu Hospital, Capital Medical University, Beijing 100053, China.; ^2^Department of Neurology, Xuanwu Hospital, Capital Medical University, Beijing 100053, China.; ^3^Department of Radiology, Dongfang Hospital of Beijing University of Chinese Medicine, Beijing 100078, China; ^4^Department of Emergency, Traditional Chinese Medicine Hospital of Yinchuan, Ningxia Hui Nationality Autonomous Region 750001, China.; ^5^Department of Neurology, Dongfang Hospital of Beijing University of Chinese Medicine, Beijing 100078, China.

**Keywords:** Herba Cistanches, anti-senescence, anti-oxidation, neuroprotection

## Abstract

The Cistanche species (“Rou Cong Rong” in Chinese) is an endangered wild species growing in arid or semi-arid areas. The dried fleshy stem of Cistanches has been used as a tonic in China for many years. Modern pharmacological studies have since demonstrated that Herba Cistanches possesses broad medicinal functions, especially for use in anti-senescence, anti-oxidation, neuroprotection, anti-inflammation, hepatoprotection, immunomodulation, anti-neoplastic, anti-osteoporosis and the promotion of bone formation. This review summarizes the up-to-date and comprehensive information on Herba Cistanches covering the aspects of the botany, traditional uses, phytochemistry and pharmacology, to lay ground for fully elucidating the potential mechanisms of Herba Cistanches’ anti-aging effect and promote its clinical application as an anti-aging herbal medicine.

## 1. Introduction

Cistanche Hoffmg. Et Link is a holoparasitic desert genus belonging to the Orobanchaceae family and has 22 species throughout the world. The Cistanche species include the perennial parasite herbs, which commonly attach itself to the roots of sand-fixing plants, such as Haloxylon ammodendron, H. persicum, Kalidium foliatum, and Tamarix plants [1]. Generally, species of the genus of Cistanche are found in arid lands and warm deserts in the northern hemisphere, such as the provinces of Xinjiang, Inner Mongolia, Gansu, Qinghai, and the Ningxia Hui Nationality Autonomous Region in China in addition to similar regions of countries such as Iran, India, and Mongolia [2].

According to the Taxonomical Index of Chinese Higher Plants, there are six Cistanche species in China [3]. However, it was confirmed that only four species and one variation of cistanche exist in China, including Cistanche deserticola Y. C. Ma, Cistanche tubulosa (Schenk) R. Wight, Cistanche salsa (C. A. Mey.) G. Beck, Cistanche salsa var. albiflora P.F. Tuet Z.C. Lou and C. sinensis G. Beck Herba [4]. Among the Cistanche species, only C. deserticola has been reordered in the Chinese Pharmacopoeia (2000 edition) [5], and C. tubulosa was added to the 2005 Chinese Pharmacopoeia as an alternative, for its similar chemical constituents, pharmacological activities and its relatively affluent resource compared to C. deserticola [6].

As a tonic, Herba Cistanches (“Rou Cong Rong” in Chinese) has been used for chronic renal disease, impotence, female infertility, morbid leucorrhea, profuse menorrhagia and senile constipation [7]. Because of its efficacy and moderate tonic character, Herba Cistanches is widely accepted and has earned the honor of “Ginseng in the deserts”.

Aging, the complex irreversible process in one’s life, is the decline in physiological body function and degradation of integrated performance. As the major risk factor for several life-threatening diseases, aging is driven by diverse molecular pathways and biochemical events that are influenced by the interplay of environmental and genetic factors [8]. Two hundred and ninety-eight genes have been collected from published works associated with aging [9]. As a biomarker of chronological aging, telomere length is linked to various aging associated diseases [10].

**Table 1:** The functions and mechanisms of Herba Cistanches extracts with anti-aging or anti-aging related effects.

Extracts	Function	Mechanisms	Refs.
Ethanol extract of Herba Cistanches	Lifespan elongation	antagonize immunosenescence, exhibit analgesic and anti-inflammatory properties, improve blood circulation, increase the weights of the seminal vesicle, prostate gland and testes, modulate serum hormone level, induct testicular steroidogenic enzymes, delay accumulation of lactic acid, improve energy storage	[23-31,36]
	Cardioprotection	reduce oxidative stress, inhibit apoptotic pathways, enhance mitochondrial ATP-GC and confer cardioprotection against ischemia/reperfusion (I/R) injury	[32,34]
	Neuroprotection	increase neuronal cell differentiation, neurite length, and synapse formation, upregulate NGF	[33]
Aqueous extract of Herba Cistanches	Lifespan elongation	inhibit cell apoptosis	[38]
	Antioxidant activity	inhibit activation of macrophage cells and nitric oxide production, scavenge free radicals	[37,39]
	Hepatoprotection	inhibit lipid peroxidation in liver microsomes	[39]
	Anti-neoplastic effect	upregulate nitric oxide synthase II expression, stimulate phagocytosis	[40]
	Memory and learning enhancement	block Aβ 1-42 amyloid deposition	[41]
	Anti-osteoporosis effect	regulate bone metabolism related genes e.g., Smad1, Smad5, TGF-β1 and TIEG1	[42-44]
	Aphrodisiac effect	alleviate spermatogenetic cell degeneration, modulate serum sex hormones levels	[45-46]
Methanol extract of Herba Cistanches	Cardioprotection	enhance mitochondrial glutathione status, decrease mitochondrial Ca^2+^ level, decrease myocardial ATP depletion, mitochondrial membrane potential and respiration rate	[47-48]
	Vasorelaxant activity	inhibit contractions induced by noradrenaline	[49]
	Hepatoprotection	inhibit D-GalN-induced death of hepatocytes, reduce TNF-α-induced cytotoxicity in L929 cells	[49]

There are several outstanding theories including mitochondrial mutation, oxidative damage, carbonyl toxification and free radical theory associated with phenomenon of aging [11, 12]. Excessive reactive oxygen species (ROS) produced during metabolism could easily lead to damage cell membranes, nucleic acids, proteins, enzymes and other biological macromolecules through peroxidation. ROS mediated modifications of delayed-rectifier and Ca^2+^ activated K^+^ channels could be linked to altered electrophysiology of neurons with clear implications in brain aging [13]. Lipid peroxidation and large amounts of harmful metabolites such as malondialdehyde (MDA) on the cell membrane, abnormality of DNA mutation or replication, together with decline of enzymes activity, consequently lead to serious damage on cell function and eventually results in senility and even death [14]. Oxidative stress has been the main cause in the etiology of many diseases, which includes Parkinson's disease (PD), Alzheimer’s disease (AD), Huntington’s disease and hepatic fibrosis.

Besides genes and oxidative stress, structural and functional defects in the immune system are closely related to aging. Immunological dysfunction with aging contributes to the increased incidence of different chronic diseases with an inflammatory component [15]. The inflammatory state is characterized by an inflammatory origin of aging given by the activation of cellular systems responsible for gene promotion and suppression such as the nuclear factor kappa B (NF-κB), sirtuins, forkhead box O and KLOTHO, which are directly or indirectly involved in cellular mechanisms of resistance to oxidative stress, apoptosis and nucleic acids repair [16].

Degenerative loss of tissue or cellular functions in the brain with a manifestation of declining logical thinking, memory and spatial abilities, is also one of the main characteristics of aging [17, 18].

Although Herba Cistanches has been used as an elixir for thousands of years, scientific research on Cistanche plants started in the 1980s. Chemical analysis indicated that various compounds, including essential oils, phenylethanoid glycosides (PhGs), iridoids, lignans, alditols, oligosaccharides and polysaccharides are the main constitutes of Cistanche plants [19]. Pharmacological research showed that extracts from Cistanche plants possess a wide spectrum of activities, such as increasing the ability to learn and memorize, treating AD, enhancing immunity, prolonging lifespan, as well as anti-aging and anti-fatigue effects [20-23]. This review aims to lay the ground for fully elucidating the potential mechanisms of Herba Cistanches’ anti-aging effect to promote its clinical application as an anti-aging herbal medicine.

## 2. Anti-aging and anti-aging related effects of Herba Cistanches extractions

Till today, the chemical constituents of Herba Cistanches include volatile compounds and more than a hundred isolated non-volatile compounds, containing PhGs, iridoids, lignans, alditols, oligosaccharides and polysaccharides [19]. Herba Cistanches is traditionally water-extracted, and recently methanol and ethanol extractions are found to be satisfactory. The extractions of Herba Cistanches with direct lifespan elongation effects or potential anti-aging properties mainly includes the ethanolic extract of aqueous extract and methanol extract. Their functions and mechanisms are found in Table 1.

### 2.1 Ethanol extract of Herba Cistanches

Ethanol extract of Cistanche tubulosa possesses significant effects in extending lifespan, which is achieved by antagonizing immunosenescence [23]. It has exhibited powerful analgesic and anti-inflammatory properties [24] and has been shown to improve blood circulation by lowering blood cholesterol levels [25]. It also could promote hair growth and relieve dandruff and scalp inflammation [26, 27]. Ethanol extract of Cistanche deserticola could increase the weights of the seminal vesicle, prostate gland and testes of castrated young rats [28, 29]. It facilitated the penis erectile response and modulated the serum hormone level to some extent [30], and also increased sex hormone levels mediated by the induction of testicular steroidogenic enzymes [31]. Herba Cistanches alcohol extract has cardioprotective and neuroprotective effects [32-34] by significantly enhancing learning and memory, and increasing neuronal cell differentiation, neurite length, and synapse formation in the mouse hippocampus [33], and reducing the oxidative stress in the reperfused myocardium, inhibiting apoptotic pathways [34]. In addition, Herba Cistanches alcohol extract possesses a sedative effect [35] and enhanced the swimming capacity of mice by decreasing muscle damage, delaying the accumulation of lactic acid and improving energy storage [36].

### 2.2 Aqueous extract of Herba Cistanches

Aqueous extract of Herba Cistanches could regulate immunity [28, 29, 37], inhibit cell apoptosis [38] and scavenge free radicals [39]. It has an anti-inflammatory effect [37, 40] and could inhibit nitric oxide (NO) production from activated RAW 264.7 macrophages [37]. Aqueous extracts of Cistanche tubulosa reduced inflammatory hyperplastic polyps and helicobacter infection in mice [40] and ameliorated the cognitive dysfunction caused by Aβ 1-42 via blocking amyloid deposition, reversing cholinergic and hippocampal dopaminergic neuronal function [41]. The water fraction of Cistanche deserticola extract could prolong the hexobarbital-induced sleeping time and reduce spontaneous motor activity [35]. Herba Cistanches aqueous extract exerts its protective effect against ovariectomized-induced bone degeneration and osteoporosis partly by regulating a few of the bone metabolism related genes e.g., Smad1, Smad5, transforming growth factor (TGF)-β1 and TGF-β inducible early gene 1 (TIEG1) [42-44]. It also alleviated the spermatogenetic cell degeneration induced by hydroxyurea [45] or Leigongteng (Radix et Rhizoma Tripterygii) [46] and modulated serum sex hormones levels to some extent [45].

**Figure 1.**
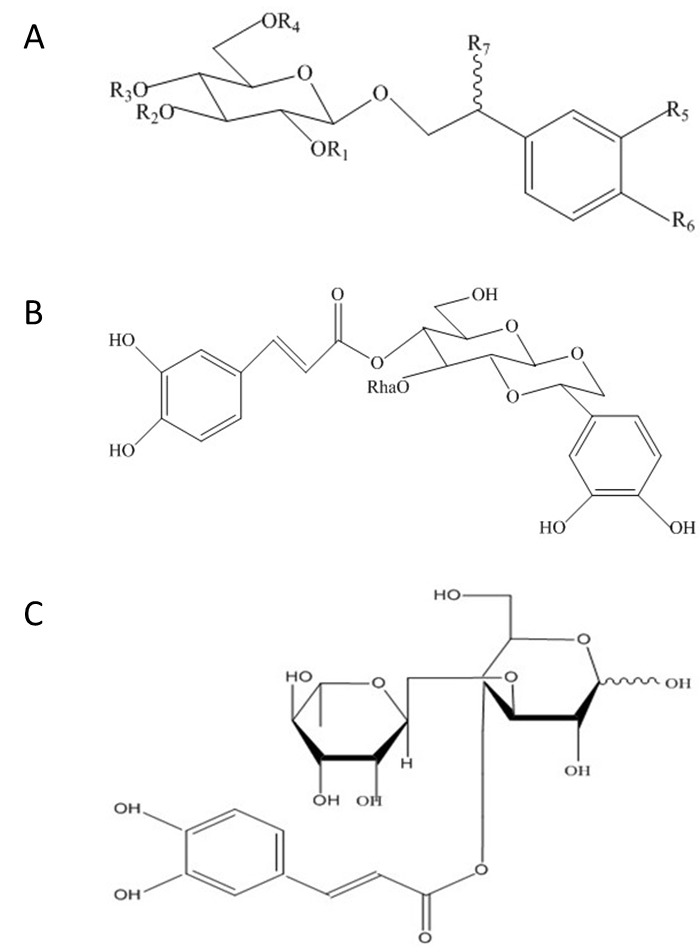
The chemical structures of PhGs (**A**) Chemical structure of compounds 1-26 in PhGs. (**B**) Chemical structure of compound 27 (Crenatoside) in PhGs. (**C**) Chemical structure of compound 28 (Cistanoside F) in PhGs.

### 2.3 Methanol extract of Herba Cistanches

One study has shown that the methanol extract of Herba Cistanches protected against myocardial ischemia/ reperfusion (I/R) injury in rats through enhancing mitochondrial glutathione status, decreasing mitochondrial Ca^2+^ level, and increasing mitochondrial membrane potential and respiration rate in rat hearts [47]. The methanolic extract from the dried stems of Cistanche tubulosa (Schrenk) R. Wight containing echinacoside and acteoside was found to show vasorelaxant activity with an inhibitory effect on contractions induced by noradrenaline in isolated rat aortic strips [48]. In addition, the methanolic extract from fresh stems of Cistanche tubulosa (Orobanchaceae) was found to have hepatoprotective effects against D-galactosamine (DGalN)/ lipopolysaccharide (LPS)-induced liver injury in mice [49].

## 3. Anti-aging and anti-aging related effects of PhGs

Pharmacological studies have shown that PhGs were the major active components of Cistanche species. PhGs have various functions, such as anti-oxidation, neuroprotection, enhancing immune and sexual function, hepatoprotection, anti-radiation, etc. PhGs were usually regarded as markers for quality evaluation of crude drugs or their corresponding formulations. To date, PhGs have been well studied and 28 PhG compounds have been isolated from Herba Cistanches (Table 2) (Fig. 1-3)

The empirical structural features of PhGs have been summarized. (1) The sugar moiety of glucose and rhamnose is connected by a Glc (3→1) Rha linkage for disaccharide glycosides; the glucose commonly links directly to an aglycone, and acoumaroyl or caffeoyl is usually located at the C4 or C6 position. (2) For becoming trisaccharide glycosides, there is an additional glucose or rhamnose at the C6 position of the inside glucose [2].

Echinacoside and acteoside, which are two major compounds of PhGs with significant pharmacological activities, can be used as quality control markers for C. deserticola and C. tubulosa. According to the Chinese Pharmacopoeia, at least 0.3% of the total contents of echinacoside and acteoside for medical application should be determined from the dried stem of C. deserticola by the high-performance liquid chromatography (HPLC) method [5].

PhGs with direct lifespan elongation effects or potential anti-aging properties mainly include the echinacoside, acteoside, isoacteoside and tubuloside which are known as the most important components of PhGs.

### 3.1 Echinacoside

Echinacoside is extracted from Cistanche tubulosa (Schrenk) R. Wight or Cistanche deserticola Y.C. Ma stems, especially in Cistanche tubulosa (Schrenk) R. Wight [56]. Its anti-aging effect has been shown in mice and cells, and it also has several anti-aging related effects, which supports its clinical application as an anti-aging drug. Its function and mechanisms are found in Table 3.

#### 3.1.1 Lifespan extension by echinacoside

Echinacoside could protect cells against aging through its anti-oxidant effect. It also induced cell cycle arrest and apoptosis in SW480 cancer cells via induction of oxidative DNA damage [57, 58]. The lifespan of wild-type worms could be extended in the presence of echinacoside. Echinacoside modulated the nuclear localization and transcriptional activities of daf-16, which fine-tuned the expression of daf-16 target genes to promote longevity and increase stress response in C. elegans [59]. Progressive mitochondrial dysfunction is considered a hallmark of aging [60, 61]. It is generally believed that premature senescence and aging caused by ROS through oxidative metabolism cause accumulation of mtDNA damage and mutations leading to the loss of fidelity in newly synthesized proteins, which ultimately impacts mitochondria physiology [62, 63]. The use of echinacoside in 1-methyl-4-phenylpyridinium ions (MPP^+^)-exposed SH-SY5Y cells has been shown to selectively reverse mitochondrial function and cell apoptosis by preventing the decrease in membrane potential of fragmented mitochondria [64]. In addition, echinacoside prevented a H_2_O_2_-induced increase of the Bax/Bcl-2 ratio by down-regulating Bax protein expression and upregulating Bcl-2 protein expression [65].

**Table 2:** Phenylethanoid glycosides from Herba Cistanche.

Active ingredient	R_1_^a^	R_2_^a^	R_3_^a^	R_4_^a^	R_5_^a^	R_6_^a^	R_7_^a^	Species^b^	Refs.
2'-Acetylacteoside (1)	Ac	Rha	Cf	H	OH	OH	H	Cd, Ct	[39, 50, 51]
Acteoside (2)	H	Rha	Cf	H	OH	OH	H	Cd, Ct	[39, 50, 51]
Cistanoside A (3)	H	Rha	Cf	Glc	Ome	OH	H	Cd, Ct	[39]
Cistanoside B (4)	H	Rha	Fr	Glc	Ome	OH	H	Cd	[52]
Cistanoside C (5)	H	Rha	Cf	H	Ome	OH	H	Cd	[52]
Cistanoside D (6)	H	Rha	Fr	H	Ome	OH	H	Cd	[52]
Cistanoside E (7)	H	Rha	H	H	Ome	OH	H	Cd	[52]
Cistanoside G (8)	H	Rha	H	H	H	OH	H	Cd	[48]
Cistanoside H (9)	Ac	Rha	H	H	OH	OH	H	Cd	[2]
Decaffeoylacteoside (10)	H	Rha	H	H	OH	OH	H	Cd, Ct	[49]
Echinacoside (11)	H	Rha	Cf	Glc	OH	OH	H	Cd, Ct	[39, 48]
Isoacteoside (12)	H	Rha	H	Cf	OH	OH	H	Cd, Ct	[39, 50, 51]
Isosyringalide-3'-α-L-rhamnopyranoside (13)	H	Rha	Cm	H	OH	OH	H	Ct	[2]
Osmanthuside (14)	H	Rha	Cm	H	H	OH	H	Cd	[53]
Salidroside (15)	H	H	H	H	H	OH	H	Cd, Ct	[49, 54]
Syringalide A-3'-α-L-rhamnopyranoside (16)	H	Rha	Cf	H	H	OH	H	Cd, Ct	[39,51]
Tubuloside A (17)	Ac	Rha	Cf	Glc	OH	OH	H	Cd, Ct	[50]
Tubuloside B (18)	Ac	Rha	H	Cf	OH	OH	H	Cd, Ct	[39, 50]
Tubuloside C (19)	Ac	TA-Rha	Cf	Glc	OH	OH	H	Ct	[2]
Tubuloside D (20)	Ac	TA-Rha	Cm	Glc	OH	OH	H	Ct	[2]
Tubuloside E (21)	Ac	TA-Rha	Cm	H	OH	OH	H	Ct	[2]
Cistantubuloside A (22)	H	Rha	Cf	Glc	H	OH	H	Ct	[49]
Cistantubuloside B_1_/B_2_(23)	H	Rha	Cm/c-Cm	Glc	OH	OH	H	Ct	[49]
Kankanoside F (24)	H	Rha	H	Glc	OH	OH	H	Ct	[48]
Kankanoside G (25)	H	Rha	H	Cf	H	OH	H	Ct	[48]
Cistantubuloside C_1_/C_2_ (26)	H	Rha	Cf	Glc	OH	OH	OH(S/R)	Ct	[55]
Crenatoside (27)								Ct	[2]
Cistanoside F (28)								Ct	[39, 48, 49]

a Cf: *trans*-caffeoyl; Cm: *trans*-coumaroyl; c-Cm: *cis*-coumaroyl; Glc: β-glucopyranose. Rha: α-L-rhamnopyranose; TA-Rha: 2’’,3’’,4’’-triacetyl-α-L-rhamnopyranose; Ac: acetyl.

b Cd: C. deserticola; Ct: C. tubulosa

PhGs containing echinacoside could enhance the activity of superoxide dismutase (SOD) significantly in the serum and brain, decrease the MDA content of liver and serum, and improve the index of spleen and thymus [66]. Cistanche deserticola containing echinacoside significantly improved the visual ability of rats by reducing the severity of the developed signs of retinopathy and cataract. The effect of Cistanche deserticola on the learning ability may be associated with differences in their redox homeostasis [67]. Cistanche deserticola may improve mucosal tissue repair by stimulating intestinal epithelial cell proliferation and preventing cell death via up-regulation of TGF-β [68].

**Table 3:** The function and mechanisms of echinacoside with anti-aging and anti-aging related effects.

Function	Mechanism	Refs.
Lifespan extension	induce cell cycle arrest and apoptosis via induction of oxidative DNA damage	[58,59,64,65,68]
	modulate nuclear localization and transcriptional activities of daf-16	
	prevent decrease in membrane potential of fragmented mitochondria	
	increase expression of the anti-apoptotic protein Bcl-2 and inhibiting caspase-3 activity	
	stimulate intestinal epithelial cell proliferation and prevent cell death via up-regulation of TGF-β	
Memory and learning enhancement	decrease P-tau phosphorylation and increase CRMP-2 expression level	[72]
Antioxidant effect	improve anti-oxidant enzymes	[39,65,74-77]
	inhibit formation of NO	
	clear all free radicals, scavenge DPPH and OH free radicals	
	protect oxidative stress-induced organ injuries by entering cells through the injured membrane, affecting the signaling pathway between ROS and the opening of Ca^2+^ channel	
Neuroprotection effect	reduce the levels of T-tau, TNF-α, and IL-1β	[78-80]
	inhibit cytochrome c release and caspase-3 activation via activating ERK pathway in neuronal cells	
	inhibit glutamate release by reducing voltage-dependent Ca^2+^ entry and suppressing protein kinase C activity	
Anti-inflammation	block TNF-α-NO and COX-II-PGE_2_ pathways	[37,74,82,83]
	scavenge NO radical	
	upregulate TGF-β1 and increase the number of Ki67(+) proliferating cells in diseased colons	
Anti-neurodegenerative effect	increase expression of GDNF and BDNF mRNA and protein, induce NTFs, inhibit apoptosis	[85]
Immunomodulatory and anti-neoplastic effects	increase intracellular oxidized guanine, 8-oxoG, and upregulate double-strand DNA break (DSB)-binding protein 53BP	[87,88]
	increase caspase 3 and cleaved PARP, upregulate G1/S-CDK blocker CDKN1B (p21) via induction of oxidative DNA damage	
Hepatoprotective effect	inhibit both ascorbic acid/Fe^2+^ and ADP/NADPH/Fe^3+^ induced lipid peroxidation	[39,49,77,88-90]
	reduce TNF-α-induced cytotoxicity	
	anti-hepatic fibrosis by reducing mRNA expression of NF-κB	
	inhibit hepatic stellate cell (HSC) activation, block conduction of TGF-β1/smad signaling pathways	
	decrease HBV replication and antigen expression	
Anti-osteoporosis effect	stimulate osteoblastic bone formation by promoting bone regeneration in cultured osteoblastic MC3T3-E1 cells	[93-96]
	increase cell proliferation, ALP activity, COL I contents, OCN levels and mineralization in osteoblasts, elevate OPG/RANKL ratio and decrease receptor activator of nuclear factor-kB ligand (RANKL) level in serum	
	promote differentiation of bone marrow mesenchymal stem cells cultured *in vitro* by increasing ZHX3 expression	
Aphrodisiac effect	increase sperm count and sperm motility and attenuate poor sperm quality and testicular toxicity in rats by up-regulating steroidogenesis enzymes including StAR, CYP11A1, 3β-HSD, 17β-HSD, CYP17A1 and CYP3A4	[31,98]
Anti-diabetic and anti-fatigue effects	suppress elevated fasting blood glucose and postprandial blood glucose levels, insulin resistance and dyslipidemia	[36,99,100]
	inhibit aldose reductase	
	enhance swimming capacity of mice by decreasing muscle damage, delay accumulation of lactic acid, and improve energy storage	

#### 3.1.2 Memory and learning enhancement effects of echinacoside

The gradual loss of cognition is one of the main characteristics of aging [69, 70]. It is recorded that PhGs containing echinacoside of Herb Cistanche could enhance the ability of learning and memorization [66]. P-tau is implicated in vascular dementia (VD) and AD because both share a common correlation with regards to vascular risk factors [71]. The glycosides of cistanche, which mainly contains echinacoside, plays a critical role in protecting hippocampal neurons in VD by decreasing P-tau phosphorylation and increasing collapsin response mediator protein-2 (CRMP-2) expression level [72]. Cistanche tubulosa glycoside capsules (CTG capsule, Memoregain®) containing mainly echinacoside had a potential to be a possible treatment option for mild to moderate AD. Memoregain® capsules are effective and safe for the treatment of moderate AD, which is in accordance with the ability of Cistanche tubulosa glycosides to inhibit excessive apoptosis of nerve cells. However, the mechanisms underlying the treatment of AD with Cistanche tubulosa glycosides are not only different from that of acetylcholinesterase inhibitors but also different from that of other types of traditional Chinese medicines. Antagonism of nerve cell apoptosis is a specific neuroprotective effect of Cistanche tubulosa glycosides [73].

#### 3.1.3 Antioxidant effect of echinacoside

Echinacoside isolated from Herba Cistanches possesses free radical scavenging properties and protects oxidative-stress-induced toxic injuries via different mechanisms. Recent studies proved the anti-oxidant activity of echinacoside, particularly in the clearing of all types of free radicals *in vivo* and *in vitro* [39, 74]. Echinacoside improved the activity of anti-oxidant enzymes and inhibited the formation of lipid peroxide, MDA and NO [66, 74-76]. It possessed free radical scavenging properties [77] and was capable of protecting against oxidative stress-induced organ injuries, by entering cells through the injured membrane, affecting the signaling pathway between ROS and the opening of the Ca^2+^ channel [66].

#### 3.1.4 Neuroprotective effects of echinacoside

Herba Cistanches containing echinacoside could improve cognitive and independent living abilities of moderate AD patients, reducing the levels of T-tau, tumor necrosis factor-α (TNF-α), and interleukin-1β (IL-1β) [78]. Transient treatment with echinacoside inhibited cytochrome c release and caspase-3 activation caused by ensuing rotenone exposure via activation of Trk-extracellular signal-regulated kinase (ERK) pathway in neuronal cells [79]. The inhibitory effect of echinacoside on evoked glutamate release was associated with reduced voltage-dependent Ca^2+^ entry and subsequent suppression of protein kinase C activity [80].

#### 3.1.5 Anti-inflammatory effects of echinacoside

Aging in humans is associated with chronic low-grade inflammation (systemic) state characterized by an increase in pro-inflammatory markers including but not restricted to TNF-α, IL-6, IL-1β, and C-reactive protein [81]. Echinacoside possesses anti-inflammatory effects through scavenging the NO radical [74, 82]. Cistanche tubulosa extract markedly attenuated inflammatory signs by blocking the TNF-α-NO and cyclooxygenase--II-prostaglandin E2 (COX-II-PGE_2_) pathways in carrageenan-induced air pouch inflammation [37]. Echinacoside protected the intestinal epithelium from inflammatory injury in DSS-induced colitis in mice by upregulating transforming growth factor (TGF)-β1 as well as increasing the number of Ki67^(+)^ proliferating cells in diseased colons [83]. In addition, echinacoside significantly alleviated the inflammatory responses induced by 6-hydroxydopamine (6-OHDA) [63].

#### 3.1.6 Anti-neurodegenerative effect of echinacoside

As echinacoside could cross the blood-brain barrier freely, it may have a promising potential in treating neurodegenerative diseases [79]; it acts as an anti-inflammatory and neuroprotective agent [65]. PhGs might exert potential inhibitory effects on microglia-involved neuroinflammation, resulting in neuroprotection in inflammation related neuronal degenerative diseases including AD and PD [65, 75, 84]. Additionally, echinacoside could increase expression of glial cell line-derived neurotrophic factor (GDNF) and brain derived neurotrophic facto (BDNF) mRNA and protein, induce neurotrophic factors (NTFs) and inhibit apoptosis [85]. Echinacoside was demonstrated to increase viability of rat pheochromocytoma PC12 cells injured by Aβ and suppress the increase in intracellular ROS triggered by Aβ. The interactions between echinacoside and amyloid-forming proteins shed light on the protection of echinacoside against amyloid fibril-induced neuronal cell death [77]. The Cistanche tubulosa extract, which contains enough echinacoside, ameliorated the cognitive dysfunction caused by Aβ 1-42 via blocking amyloid deposition especially in hippocampal areas [41].

#### 3.1.7 Immunomodulatory and anti-neoplastic effects of echinacoside

Extensive evidence exists indicating that aging in an organism is characterized with immune deficiency [86]. Echinacoside could be used as a specific immunostimulatory adjuvant against colorectal cancer [58]. Echinacoside caused a significant increase of intracellular oxidized guanine, 8-oxoG [87], a dramatic upregulation of the double-strand DNA break (DSB)-binding protein 53BP, induced cell cycle arrest and apoptosis, and significantly increased active caspase 3 and cleaved poly ADP-ribose polymerase (PARP). It upregulated the G1/S-CDK blocker CDKN1B (p21) in SW480 cancer cells via induction of oxidative DNA damage [58].

**Table 4:** The functions and mechanisms of acteoside with anti-aging and anti-aging related effects.

Function	Mechanism	Refs.
Lifespan extension	inhibit hepatic apoptosis	[49,101,102]
Memory and learning enhancement	promote NGF and its neuronal actions, increase TrK A expression, upregulate NGF	[33,103-105]
	inhibit acetylcholine esterase and increase the activities of antioxidant enzymes	
	increase activity of GSH-Px, T-SOD, TChE and protein contents, and decrease MDA content	
	increase neurons and nissl bodies in the hippocampus, promote NGF and TrkA expression, decrease the content of nitric oxide, activity of nitric oxide synthase and expression of caspase-3 protein	
Antioxidant effect	scavenge NO radical and DPPH radical	[38,65,103,106,107]
	decrease activity of nitric oxide synthase	
	inhibit both ascorbic acid/Fe^2+^ and ADP/NADPH/Fe^3+^ induced lipid peroxidation in rat liver microsomes	
Neuroprotective effect	increase neurons and nissl bodies in the hippocampus	[41,103,104,108,109]
	inhibit rotenone-induced α-synuclein, caspase-3 upregulation and MAP-2 downregulation	
	block amyloid deposition, reverse cholinergic and hippocampal dopaminergic neuronal function	
	improve SK-N-SH cell morphology, enhance cell survival rate, decrease cell LDH release rate and expression of phosphorylated tau proteins at p-Ser 199/202 and p-Ser 404 sites, up-regulate the expression of non-phosphorylated tau proteins at Ser 202 site and Ser 404 sites	
Anti-inflammatory effect	scavenge NO radical	[74]
Immunomodulatory and anti-neoplastic effects	inhibit basophilic cell-derived immediate-type and delayed-type allergic reactions	[110,111]
	down-regulate expressions of the CCL1, CCL2, CCL3, CCL4, FCER1A and NFATC1 genes, inhibit MAPK pathway, and decrease JNK phosphorylation	
Hepatoprotective effect	antioxidative, immunoregulatory, regulate hepatic apoptosis	[49,50,90,96,102]
	inhibit TNF-α-mediated hepatic apoptosis and subsequent necrosis in DGalN/LPS-induced liver failure	
	scavenge free radicals, inhibit lipid peroxidation, protect hepatic membranes	
	block the TGF-β1/smad signaling pathway and inhibit the activation of HSC	
	inhibit D-GalN-induced death of hepatocytes and reduce TNF-α-induced cytotoxicity in L929 cells	
	block P450-mediated bioactivation	
Anti- hypercholesterolemia and anti-diabetic effects	enhance mRNA expressions of apolipoprotein B, VLDL receptor, and cytochrome P450 SCC in HepG2 hepatocytes, in diet-induced hypercholesterolemia mice	[25,36]
	improve glucose tolerance in starch-loaded mice	

#### 3.1.8 Hepatoprotection effects of echinacoside

Echinacoside exhibited significant inhibition on both ascorbic acid/Fe^2+^ and ADP/NADPH/Fe^3+^ induced lipid peroxidation in rat liver microsomes, which were more potent than α-tocopherol of caffeic acid [39]. It also inhibited D-GalN-induced death of hepatocytes and reduced TNF-α-induced cytotoxicity in L929 cells [49]. It was reported that PhGs have significant anti-hepatic fibrosis effects by reducing NF-κB RNA levels [88, 89]. Echinacoside possesses an anti-hepatic fibrosis effect by inhibiting hepatic stellate cell (HSC) activation and the TGF-β1/smad pathway (increasing the mRNA level and protein expression of smad7, and decreasing the mRNA levels of smad2, smad3 and the protein expression of smad2, phospho-smad2, smad3, phospho-smad3) [89]. Echinacoside could also provide a definite protective effect against acute hepatic injury by ameliorating histopathological damage of the liver and the number of apoptotic hepatocytes, which was accompanied by the reduction of serum alanine aminotransferase (ALT), aspertate aminotransferase (AST) levels and hepatic MDA content as well as ROS production, and the restoration of hepatic SOD activity and glutathione (GSH) content [77]. Echinacoside also has a strong effect against hepatitis B virus (HBV) replication and antigen expression [90].

#### 3.1.9 Anti-osteoporosis effects of echinacoside

Osteoporosis has already become one of the leading threats for the health of the aging population [91, 92]. Echinacoside, like estrogen, has a stimulatory effect on osteoblastic bone formation whereby it promotes bone regeneration in cultured osteoblastic MC3T3-E1 cells. It was effective and safe in treating ovariectomy (OVX)-induced osteoporosis by increasing cell proliferation, alkaline phosphatase (ALP) activity, collagen type I (COL I) contents, osteocalcin (OCN) levels and mineralization in osteoblasts, decreasing receptor activator of nuclear factor-??B ligand (RANKL) level and elevating the osteoprotegerin (OPG)/RANKL ratio in serum [93-95]. In addition, echinacoside could promote the differentiation of bone marrow mesenchymal stem cells *in vitro*, and the mechanism may be correlated with the increase in the zinc fingers and homeoboxes 3 (ZHX3) expression [96].

#### 3.1.10 Aphrodisiac effects of echinacoside

There are different opinions about the safety of Herba Cistanches treatment for diseases involving the male reproductive system. Some studies showed that Herba Cistanches displayed cytotoxic effects on the male reproductive system, thus it may not be appropriate for therapies seeking to improve the function of the male reproductive system [97]. Other studies demonstrated that Echinacoside could increase sperm count and sperm motility, and attenuate poor sperm quality and testicular toxicity in rats through the up-regulation of steroidogenesis enzymes including steroidogenic acute regulatory protein (StAR), cytochrome P450 cholesterol side-chain cleavage enzyme (CYP11A1), 3β-hydroxysteroid dehydrogenase (3β-HSD), 17β-HSD, CYP17A1 [98] and CYP3A4 [31].

#### 3.1.11 Anti-diabetic and anti-fatigue effects of echinacoside

Cistanche tubulosa could significantly suppress elevated fasting blood glucose and postprandial blood glucose levels, improve insulin resistance and dyslipidemia, and suppress body weight loss in db/db mice [99]. One study showed that echinacoside had potent aldose reductase inhibitory activity [100]. The phenylethanoid-rich extract of Cistanche deserticola Y.C. Ma containing echinacoside as its major constituent played an important role in antifatigue activity through enhancing the swimming capacity of mice by decreasing muscle damage, delaying the accumulation of lactic acid, and by improving energy storage [36].

### 3.2 Acteoside (Verbascoside)

Acteoside, also known as verbascoside or orobanchin, is another major active phenylethanoid glycoside present in Herba Cistanches. Its functions and mechanisms are summarized in Table 4.

#### 3.2.1 Lifespan extension by acteoside

There are no direct reports about lifespan extension by acteoside. Previous studies have indicated that acteoside significantly improved cell viability via anti-apoptotic effects. Acteoside exhibits a significant inhibitory effect on hepatic apoptosis [49, 101, 102]. In addition, acteoside could improve learning and memory in a mouse model of senescence induced by a combination of D-galactose and AlCl3 [103]. It also improved the behavior in senescence-accelerated OXYS rats [67].

#### 3.2.2 Memory and learning enhancement effects of acteoside

Acteoside has been shown to have significant protective effects on learning and memory impairment in a mouse scopolamine-induced amnesia model through the increase in the activities of GSH-Px, T-SOD, total cholinesterase (TChE), and decreasing MDA content [103]. The mechanisms of memory enhancement of acteoside were partly due to inhibition of acetylcholine esterase and elevation of antioxidant enzymes [33]. Acteoside could decrease the activity of NO synthase and the expression of caspase-3 protein [104]. It also could promote the release of nerve growth factor (NGF) and neuronal actions, including neurite outgrowth and synapse formation, and increasing tropomycin receptor kinase A (TrkA) expression [105]. One clinic trial showed that Herba Cistanches containing acteoside could improve cognitive and independent living abilities of moderate AD patients [77].

#### 3.2.3 Antioxidant effect of acteoside

Acteoside protects the cell from oxidative stress and scavenging of free radicals. As an antioxidant, acteoside could not only scavenge radical oxygen, such as NO radical and 1,1-diphenyl-2-picrylhydrazyl (DPPH) radical [65, 106], but also decrease the activity of NO synthase [103]. Acteoside showed stronger free radical scavenging activities than α-tocopherol on DPPH radical and xanthine/xanthine oxidase generated superoxide anion radical O^2-^, and exhibited significant inhibition on both ascorbic acid/Fe^2+^ and ADP/NADPH/Fe^3+^ induced lipid peroxidation in rat liver microsomes [38]. The numbers of phenolic hydroxyl groups of phenylpropanoid glycosides are directly related to their scavenging activities. The scavenging activities are likely related to the odihydroxy group of phenylpropanoid glycosides as well [107].

#### 3.2.4 Neuroprotective effect of acteoside

Recent studies indicated that acteoside could exhibit neuroprotective capabilities [104, 105]. Acteoside could increase the number of neurons and nissl bodies in the hippocampus [104]. Acteoside significantly attenuated Parkinsonism symptoms by inhibiting rotenone-induced α-synuclein and caspase-3 upregulation, and microtubule-associated protein 2 downregulation in PD rats [108]. Acteoside ameliorated the cognitive dysfunction caused by Aβ 1-42 via blocking amyloid deposition, reversing cholinergic and dopaminergic neuronal function [41]. In addition, acteoside has significant protective effect on a cellular model of AD induced by okadaic acid through improving SK-N-SH cell morphology, enhancing cell survival rate, decreasing cell lactate dehydrogenase release rate and the expression of phosphorylated tau proteins at the p-Ser 199/202 and p-Ser 404 sites, and up-regulating the expression of non-phosphorylated tau proteins at the Ser 202 site and Ser 404 sites [109].

#### 3.2.5 Anti-inflammatory effect of acteoside

Acteoside had an anti-inflammatory effect against D-galactosamine/ lipopolysaccharide-induced hepatitis in mice [109], which was possibly related to its NO radical-scavenging activity [74].

#### 3.2.6 Immunomodulatory and anti-neoplastic effects of acteoside

Acteoside is a potent immunostimulant with extensive effects on immune organs, immune cells and immune factors. It could inhibit basophilic cell-derived immediate-type and delayed-type allergic reactions. It was reported that acteoside inhibited the release of β-hexosaminidase and Ca^2+^ influx from immunoglobulin E-mediated RBL-2H3 cells. It inhibited histamine release, production of TNF-α and IL-4 in human basophilic (KU812) cells [110]. The anti-allergy effects of acteoside were due to downregulation of the expressions of the chemokine ligand (CCL) 1, CCL2, CCL3, CCL4, Fc fragment of IgE, high affinity I, receptor for alpha polypeptide (FCER1A), nuclear factor of activated T cell, cytoplasmic, and calcineurin-dependent 1 (NFATC1) genes and inhibition of the mitogen-activated protein kinase (MAPK) pathway through decreased C-jun N terminal kinase (JNK) phosphorylation [111]. Acteoside also exhibited inhibitory effects on the proliferation of the prostate cancer PC-3 cell line, which was roughly double the potency afforded by echinacoside [86].

#### 3.2.7 Hepatoprotective effect of acteoside

The mode of action of acteoside in hepatic protection is at least in part related to its antioxidative, immunoregulatory properties and its ability to regulate hepatic apoptosis [96, 102]. Acteoside could effectively inhibit TNF-α-mediated hepatic apoptosis and the subsequent necrosis in DGalN/LPS-induced liver failure. The protective effect of acteoside on immunological liver injury may be due to its ability to scavenge free radicals, inhibit lipid peroxidation, protect hepatic membranes, and restore the balance of Th1/Th2 and Bax/Bcl-2 [102]. The protective effects against carbon tetrachloride was possibly related to the acteoside’s ability to block the P450-mediated bioactivation and scavenge free radicals during liver injury [50]. Acteoside also inhibited DGalN-induced death of hepatocytes and reduced TNF-α-induced cytotoxicity in L929 cells [49]. Acteoside may be a potential herbal medicine for the treatment of liver fibrosis because of its ability to block the TGF-β1/smad signaling pathway and inhibit the activation of hepatic stellate cell [90].

#### 3.2.8 Anti-hypercholesterolemia and anti-diabetic effects of acteoside

Acteoside, acquired from the aqueous ethanol extract of the roots of Cistanche tubulosa, was involved in regulating the hypocholesterolemic activity through enhancing the mRNA expressions of apolipoprotein B, very low-density lipoprotein (VLDL) receptor, and cytochrome P450 SCC in HepG2 hepatocytes of hypercholesterolemia mice [25]. Acteoside was also found to significantly improve glucose tolerance in starch-loaded mice [36].

**Table 5:** The function and mechanisms of isoacteoside with anti-aging related effects.

Function	Mechanism	Refs.
Antioxidant effect	scavenge free radical such as NO radical	[39,74,83]
Hepatoprotective effect	inhibit both ascorbic acid/Fe^2+^ and ADP/NADPH/Fe^3+^ induced lipid peroxidation in rat liver microsomes	[39,49]
	reduce TNF-α-induced cytotoxicity	
Neuroprotective effect	inhibit microglia-involved neuroinflammation	[83]

### 3.3 Isoacteoside

Isoacteoside is one of the phenylethanoid compounds isolated from Herba Cistanches. Previous studies mainly focused on its anti-oxidant, anti-apoptotic, neuroprotective and hepatoprotective effects. Isoacteoside showed strong free radical scavenging activities [39, 74, 83] and hepatoprotective activity against either the radical generator carbon tetrachloride (CCl (4)) or specific liver toxin DGalN [49, 50]. The mechanisms of hepatoprotective effects were also related to its inhibitory effects on both ascorbic acid/Fe^2+^ and ADP/NADPH/Fe^3+^ induced lipid peroxidation in rat liver microsomes [39]. It could also reduce TNF-α-induced cytotoxicity in L929 cells [49]. In addition, isoacteoside was found to have potential inhibitory effects on microglia-involved neuroinflammation [83]. Its function and mechanisms are shown in Table 5.

### 3.4 Pharmacological comparison of echinacoside, acteoside and isoacteoside

Antioxidative effects were found to be potentiated by an increase in the number of phenolic hydroxyl groups in the molecule [39]. The anti-oxidative activity of PhGs is related to steric hindrance, the presence of 2-acetyl on the middle glucopyranose, the location and number of phenolic hydroxyls in the molecule. Additionally, it may be related to the α-, β-unsaturated ketone of phenyl-2-propenoyl [112]. Moreover, there is a certain dose-effect relationship *in vivo* with respect to the hepatoprotective effects of echinacoside, acteoside and isoacteoside [49]. The hepatoprotective or inhibition of HSC activity of acteoside is better than that of echinacoside, which may attribute to the presence of steric hindrance [90].

## 4. Polysaccharides from Herba Cistanches

Polysaccharides are compounds from the water or ethanol extract of Herba Cistanches. Studies found that polysaccharides from Cistanche deserticola have anti-hyperglycemic and hypolipidemic effects [99], immunological activity [113], proliferative effect on lymphocytes [114], antioxidant potential *in vitro* [115], and hepatoprotective activity [116]. They are potent for the stimulation of both T- and B-cell proliferation [117]. Their origin, functions, and mechanisms are found in Table 6.

## 5. Extractions or bioactive components with potential anti-aging effects obtained from other species of Cistanches

In recent years, the wild Cistanche deserticola Y.C. Ma and Cistanche tubulosa (Schrenk) R. Wight are close to being extinct due to over-harvesting, and thus need protection in China. Due to the deficiency of the natural resources of the official Herba Cistanche, other species of this genus such as the Cistanche salsa (C. A. Mey.) G. Beck and Cistanche violacea Desf. (Orobanchaceae) are also used as substitutes in many areas. Other Cistanches species have demonstrated potential anti-aging effects.

### 5.1 Cistanche Salsa

Cistanche salsa (C.A. Mey.) G. Beck, found in the provinces of northwest China, is also used in some regions due to the resource shortage. It was found that Cistanche deserticola Y.C. Ma and Cistanche salsa (C.A. Mey.) G. Beck were used as Herba Cistanches in traditional Chinese medicine according to the ancient herbal records. Its active ingredients, functions and mechanisms are summarized in Table 7.

#### 5.1.1 Anti-aging related effects of Cistanche Salsa extractions

The extract of Cistanche salsa significantly suppressed loss of bone weight in ovariectomized mice [118]. It may be a potential therapeutic candidate for benign prostate hyperplasia (BPH) owing to its ability to decrease prostate weight, serum dihydrotestosterone (DHT) concentration, and mRNA expression of 5α-reductase type 1 and type 2 in prostate tissue of BPH-induced rats. It also suppressed cell proliferation by regulating the expression levels of inflammatory-related proteins (inducible NO synthase and cyclooxygenase 2) and apoptosis-associated proteins (caspase-3 and Bcl-2 family proteins) [119].

**Table 6:** Active ingredients, functions and mechanisms of Polysaccharides from Herba Cistanches with anti-aging related effects.

Function	Active ingredient	Mechanism	Refs.
Immunological activity	ACDP-2	stimulate immune response	[114]
	Cistanche Deserticola polysaccharide (CDPS)	stimulate the division of thymus lymphocyte, promote thymus intracellular calcium delivering	[113]
	CDA-1A	stimulate B cell proliferation	[117]
	CDA-3B	stimulate both T and B cell proliferation	[117]
Hepatoprotective effect	CDP-C	antioxidant, promote viability of HepG2 cells, attenuate microvesicular steatosis and mild necrosis, reduce the contents of MDA and TG	[116]
Antioxidant activity	Cistanche tubulosa polysaccharide (CTP)	scavenge DPPH, OH and ABTS radical	[115]
Anti-diabetic effect	Cistanche tubulosa polysaccharide (CTP)	suppress elevated fasting blood glucose and postprandial blood glucose levels, insulin resistance and dyslipidemia suppress body weight loss in db/db mice	[99]

#### 5.1.2 Anti-aging and anti-aging related effects of PhGs

Like Cistanche deserticola and Cistanche tubulosa, PhGs are the major active components of Cistanche Salsa. It was reported that PhGs from Cistanches salsa could prevent cell apoptosis [120] and protect dopaminergic neurons against dopamine neurotoxicity induced by 1-methyl-4-phenyl-1,2,3,6-tetrahydropyridine (MPTP) in C57 mice [121]. Echinacoside, acteoside and tubuloside are major compounds of PhGs with significant pharmacological activities.

#### 5.1.3 Anti-aging and anti-aging related effects of echinacoside

##### 5.1.3.1 Lifespan extension by echinacoside

Echinacoside could improve ROS degradation [122] and retard human fibroblastic cellular senescence through protecting cells from DNA damage by triggering cells in the G1 phase to enter the S phase and G2 phase. In addition, echinacoside could protect neuronal cells from apoptosis [123] by maintaining mitochondrial function, decreasing the generation of ROS, increasing the expression of the anti-apoptotic protein Bcl-2 and inhibiting caspase-3 activity through an antioxidative mechanism [124].

##### 5.1.3.2 Anti-inflammatory effect of echinacoside

There was some evidence to support the anti-inflammatory effect of echinacoside [125]. Echinacoside showed anti-inflammatory property, characterized by a significant reduction in the inflammatory markers, including myeloperoxidase, extracellular nucleosomes, high-mobility group box 1, and inflammatory cytokines in the plasma of D-galactosamine and lipopolysaccharide-induced acute liver injury, which may be important mechanisms related to its protective effect [126].

**Table 7:** Active ingredients, functions and mechanisms of Cistanche Salsa with anti-aging and anti-aging related effects.

Active ingredient	Function	Mechanism	Refs.
Ethanol extract of Cistanche Salsa	Anti-osteoporosis effect	suppress bone weight loss	[118]
	Anti-proliferative effect	decrease prostate weight, serum dihydrotestosterone concentration, and mRNA expression of 5α-reductase type 1 and type 2, regulate the expression levels of inflammatory-related proteins and apoptosis-associated proteins	[119]
Phenylethanoid glycosides (PhGs)	Neuroprotective effect	prevent cell apoptosis, protect dopaminergic neurons against dopamine neurotoxicity induced by MPTP	[120-121]
Echinacoside	Lifespan extension	protect cells from DNA damage, trigger cells in the G1 phase to enter the S phase and G2 phase, improve ROS degradation	[122-124]
		protect neuronal cells from apoptosis	
		maintain mitochondrial function, decrease the generation of ROS, increase the expression of the antiapoptotic protein Bcl-2 and inhibit caspase-3 activity	
	Anti-inflammatory effect	reduce inflammatory markers, including myeloperoxidase, extracellular nucleosomes, high-mobility group box 1, and inflammatory cytokines	[126]
	Neuroprotective effect	inhibit caspase-3 and caspase-8 activation in cerebellar granule neurons	[127-130]
		reduce ROS production, attenuate neurotoxicity mitochondrial dysfunction and inflammatory responses induced by 6-OHDA	
		suppress expression of apoptotic genes	
		inhibit generation of MPP^+^-induced ROS	
		decrease striatal extracellular levels of DA, DOPAC and HVA	
	Anti-neurodegenerative effect	attenuate neurotoxicity mitochondrial dysfunction and inflammatory responses	[123,125,127-128]
		suppress dopaminergic neuron loss caused by MPP^+^ or MPTP	
		maintain dopamine content and dopamine metabolite content	
		increase striatal dopamine and dopamine metabolite levels	
		inhibit apoptosis and activation of microglia and astrocytes in the substantia nigra	
		regulate cytokines such as p38 MAPK and NF-κB p52 subunit	
	Anti-oxidant effect	decrease generation of ROS and protect oxidative-stress-induced toxic injuries	[124]
	Anti-neoplastic effect	inhibit prostate cancer cell proliferation	[132,133]
		modulate MAPK activity	
Acteoside	Skin-protective effect	enhance scavenging activity of ROS, decrease Bax/Bcl-2 ratio and downregulate activity of pro caspase-3	[135]
		modulate the MAPK signaling pathway	
	Neuroprotective effect	inhibit neuronal death induced by MPP^+^ and glutamate	[134]
Tubuloside B	Neuroprotective effect	inhibit cell apoptosis, attenuate MPP^+^ induced cytotoxicity, DNA fragmentation, and intracellular accumulation of ROS, anti-oxidative stress effects, maintenance of mitochondria function, decrease of concentration of free intracellular calcium, inhibition of caspase-3 activity	[136,137]
(2E,6R)-8-Hydroxy-2,6-dimethyl-2-octenoic acid [(R)-HDOA]	Anti-osteoporosis effect	decrease bone weight and mechanical strength	[133]

##### 5.1.3.3 Neuroprotective effect of echinacoside

Echinacoside significantly inhibited caspase-3 and caspase-8 activation in cerebellar granule neurons [127], reduced ROS production, attenuated neurotoxicity mitochondrial dysfunction and inflammatory responses induced by 6-OHDA [128]. In addition, echinacoside significantly improved neuroblastoma cell survival by inhibiting the generation of MPP^+^-induced ROS by suppressing the expression of apoptotic genes (ATF3, CHOP and SCNA) [129]. Echinacoside could protect dopaminergic neurons through significantly decreasing striatal extracellular levels of dopamine, 3,4-dihydroxy-phenylacetic acid (DOPAC) and catabolites homovanillic acid (HVA) [130].

##### 5.1.3.4 Anti-neurodegenerative effect of echinacoside

Modern pharmacological studies determined that echinacoside significantly improves learning and memory [125]. Mitochondrial dysfunction and inflammatory responses are involved in the mechanism of cell damage in neurodegenerative diseases. One study demonstrated that echinacoside significantly attenuated neurotoxicity mitochondrial dysfunction and inflammatory responses [128]. It also significantly suppressed dopaminergic neuronal loss caused by MPP^+^ [127] or MPTP and maintained dopamine content and dopamine metabolite content compared with those measured in mice with MPTP-induced damage [127]. In addition, echinacoside inhibited the apoptosis and activation of microglia and astrocytes in the substantia nigra, which suggested its involvement in the regulation of neuro-inflammation through cytokines such as p38 MAPK and the NF-κB p52 subunit [123].

##### 5.1.3.5 Antioxidant effect of echinacoside

It was reported that echinacoside with antioxidative properties could inhibit apoptosis by decreasing the generation of ROS and protecting from oxidative-stress-induced toxic injuries [124]. Echinacoside could also provide a protective effect against acute hepatic injury caused by CCl(4) in rats, which may be associated with its antioxidative effect [131].

##### 5.1.3.6 Anti-neoplastic effect of echinacoside

One study showed that echinacoside exhibited an inhibitory effect on prostate cancer cell proliferation [132]. Echinacoside also markedly repress the proliferation of pancreatic adenocarcinoma cells by inducing the production of ROS and the perturbation of the mitochondrial membrane potential thus triggering apoptosis. Furthermore, it was elucidated that echinacoside inhibits tumor cell growth through modulating MAPK activity [133].

#### 5.1.4 Anti-aging and anti-aging related effects of acteoside

One study reported that the strength of the antioxidative activity of acteoside was better than that of echinacoside in Cistanche salsa. Acteoside from Cistanche salsa was shown to inhibit neuronal cell death induced by MPP^+^ and glutamate [134]. Acteoside could protect the cells from X-ray induced damage through enhancing the scavenging activity of ROS, decreasing the Bax/Bcl-2 ratio, downregulating the activity of pro caspase-3, and modulating the MAPK signaling pathway [135].

#### 5.1.5 Anti-aging and anti-aging related effects of tubuloside B

Tubuloside B is one of the phenylethanoid compounds isolated from Cistanche salsa. It has neuroprotective effects against MPP^+^ toxicity by inhibiting MPP^+^ induced apoptosis and oxidative stress [136], and against TNF-α-induced apoptosis in SH-SY5Y cells via anti-oxidative stress effects, maintenance of mitochondria function, decrease of concentration of free intracellular calcium and inhibition of caspase-3 activity [137].

The antioxidative effect was found to be potentiated by an increase in the number of phenolic hydroxyl groups in the molecule. The sequence of the strength of the antioxidative activity of the four components is as follows: Acteoside ≥ Tubuloside B ≥ Isoacteoside > Echinacoside [127].

#### 5.1.6 Anti-aging related effects of monoterpene in Cistanche Salsa

(2E,6R)-8-Hydroxy-2,6-dimethyl-2-octenoic acid [(R)-HDOA], a novel monoterpene from Cistanche salsa, suppressed the decrease of bone weight and the mechanical strength in ovariectomized mice, and was found to be an anti-osteoporotic compound [133].

### 5.2 Cistanche violacea Desf.

Cistanche violacea, an endemic shrub in Northern Africa, is a holoparasitic plant found on Chenopodiaceae and Limoniastrum. The anti-inflammatory activity of C. violacea ethanol extract probably inhibited arthritic activity and lysosomal release, which might be influenced by the synergistic action of iridoids, phenylethanoids glycosides and apigenin compounds [138].

## 6. Conclusion

Contemporary pharmacological studies have gradually validated Herba Cistanches’ traditional uses. The crude extracts and compounds such as Phenylethanoid glycosides, acteoside, isoacteoside, echinacoside and tubuloside B from the stem have been shown to possess multiple medical functions especially for bone metabolism, the reproductive system, immune system and central nervous system in *in vitro* and *in vivo* studies. In view of its evident therapeutic efficiency and economic advantages, it may be beneficial to develop the active ingredients of Herba Cistanches as new pharmaceutical agents for the treatment of various diseases.

However, the exact chemical compound responsible for the observed pharmacological effects is still not known. The monomeric constituents of Herba Cistanches should therefore be investigated further to gain a better understanding of the individual components’ pharmacological mechanisms. In addition, clinical studies in humans are urgently needed to confirm the claims of conventional phytotherapy. The side effects and potential interactions between Herba Cistanches and other synthetic drugs should also be further investigated. These may be important future challenges in drug discovery.
